# Observation of Collective Photoswitching in Free‐Standing TATA‐Based Azobenzenes on Au(111)

**DOI:** 10.1002/anie.202003797

**Published:** 2020-07-27

**Authors:** Talina R. Rusch, Alexander Schlimm, Nicolai R. Krekiehn, Tobias Tellkamp, Šimon Budzák, Denis Jacquemin, Felix Tuczek, Rainer Herges, Olaf M. Magnussen

**Affiliations:** ^1^ Institute of Experimental and Applied Physics Christian Albrechts University Kiel Germany; ^2^ Institute of Inorganic Chemistry Christian Albrechts University Kiel Germany; ^3^ Otto Diels Institute of Organic Chemistry Christian Albrechts University Kiel Germany; ^4^ Department of Chemistry Faculty of Natural Sciences Matej Bel University Banska Bystrica Slovakia; ^5^ CEISAM Lab—UMR 6230— CNRS/University of Nantes Nantes France

**Keywords:** cooperative effects, functional self-assembled monolayers, in situ STM, isomers, photoisomerization

## Abstract

Light‐induced transitions between the *trans* and *cis* isomer of triazatriangulenium‐based azobenzene derivatives on Au(111) surfaces were observed directly by scanning tunneling microscopy, allowing atomic‐scale studies of the photoisomerization kinetics. Although the azobenzene units in these adlayers are free‐standing and spaced at uniform distances of 1.26 nm, their photoswitching depends on the isomeric state of the surrounding molecules and, specifically, is accelerated by neighboring *cis* isomers. These collective effects are supported by ab initio calculations indicating that the electronic excitation preferably localizes on the *n*–π* state of *trans* isomers with neighboring *cis* azobenzenes.

## Graphical abstract

**Photoswitching** in a well‐ordered self‐assembled monolayer of freestanding azobenzene derivatives was studied on the molecular scale with in situ scanning tunneling microscopy, infrared absorption spectroscopy, and ab initio calculations, revealing cooperative effects. Although the intermolecular distances prevent direct contact, *trans*–*cis* photoisomerization is enhanced by *cis* neighbors because the light‐induced excitation is localized.

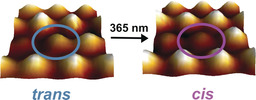

The development of photocontrolled surfaces consisting of highly ordered molecular switches is an important step towards integrating machine‐like functions in solid‐state devices and thus has received much interest. Self‐assembled monolayers (SAMs) of azobenzene and its derivatives have been intensely investigated for this purpose.^1^, ^2^, ^3^, ^4^ Switching between the *trans* and *cis* states of adsorbed azobenzene derivatives were not only identified by various spectroscopic methods but also at the nanoscale using scanning probe microscopy.^5^, ^6^, ^7^, ^8^, ^9^, ^10^, ^11^ While in densely packed aliphatic azobenzene, SAMs switching is prevented;^12^ densely packed aromatic azobenzene SAMs exhibited reversible, collective photoswitching.^7^, ^13^, ^14^, ^15^ The latter is surprising and was attributed largely to steric effects, caused by the rigidity of the molecule and stabilization of the *cis* isomer by intermolecular interactions.^7^, ^16^, ^17^ Furthermore, excitonic coupling was proposed to lead to cooperativity in diluted aliphatic azobenzene SAMs.^18^

Herein, we report on collective photoswitching in adlayers of freestanding well‐separated azobenzene derivatives, bound to Au(111) surfaces via a triazatriangulenium (TATA) platform (Figure 1 a). In the employed molecule, ((*E*)‐12*c*‐ [(4‐4‐[(4‐methoxyphenyl)diazenyl]phenyl‐2,6,7‐trioxabicyclo[2.2.2]octane‐1‐yl)ethynyl]‐4,8,12‐tri‐*n*‐octyl‐4,8,12‐triazatriangulene (compound **1**), the azobenzene switch is linked to the TATA platform via an isolating trioxabicyclo [2.2.2] unit.

**Figure 1:**
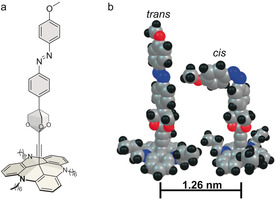
a) Studied compound **1**. b) Illustration of a *cis* and *trans* isomer, placed at the distance of neighboring adsorbates in the **1** adlayer. The depicted molecular geometries show the van der Waals spheres and were calculated using DFT (M062X‐D3/def2‐TZVP).^19^

The molecules adsorbed intact on the surface in the form of a well‐ordered hexagonal (√19×√19) *R*23.4° superstructure with a nearest neighbor distance of (12.6±0.5) Å, and they were reversibly switchable in the SAM according to spectroscopic data (Figure 3 f; Supporting Information, Figure S5).^19^ The absence of steric effects is expected because of the open arrangement of the molecules on the surface (Figure 1 b) and is supported by quantitative studies of mixed adlayers of **1** and TATA derivatives with different vertical groups, which find random distributions of the adsorbed molecules.^20^ The isolating unit leads to a weak electronic coupling between the azobenzene unit and the Au surface, resulting in a high stability of the *cis* state against thermal back‐isomerization (*t*_1/2_=61.1 h at 290.5 K). This enables direct observations of the switching process via scanning tunneling microscopy (STM).

In the initial state, obtained immediately after preparation, **1** SAMs on Au(111) showed a well‐ordered hexagonal adlayer in the STM images, in which all molecules appear equal (Figure S3). Upon irradiation of the sample with UV light (365 nm, 6 μW cm^−2^), the lateral arrangement of the molecules remained unchanged, but the appearance of the adlayer changed distinctly: instead of a single molecular species, two type of molecules with different apparent heights were visible (Figures 2 a,b). These height changes can be reversed by irradiation with blue light (440 nm), indicating that they correspond to the reversible switching of the molecules, rather than to irreversible light‐induced reactions, as for example, photochemical cleavage.^21^ The molecules with lower apparent height became more numerous with UV irradiation time (Figures 2 c,d) and can thus be assigned to the *cis* isomer; the higher molecules therefore correspond to the *trans* isomer. Without further irradiation, the spatial distribution of the isomers did not change, indicating that the switching is not induced by the STM imaging. Furthermore, significant changes were only observed in experiments, where the tip was withdrawn from the surface during irradiation. In the sequences of STM images recorded during irradiation only minor *trans*–*cis* isomerization was observed, which we attribute to shading by the STM tip.

**Figure 2:**
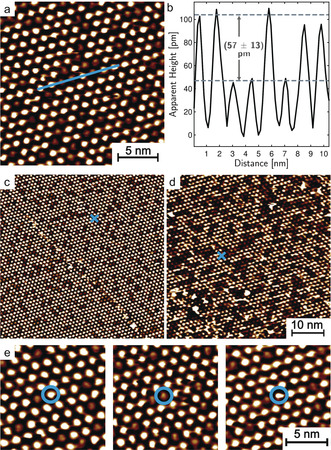
STM images of SAMs of **1** on Au(111) after irradiation with 365 nm at 6 μW cm^−2^ a,c) after *t*_1_=28 min, and d) after *t*_2_=71 min of irradiation. Blue crosses mark the same area on the surface. b) Cross‐section taken along the blue line in (a). e) Series of STM images, showing the same surface area after 28, 32, and 38 min UV irradiation, respectively, illustrating light‐induced dynamic changes between the *trans* and *cis* state. In all experiments, the tip was withdrawn from the surface during irradiation.

Observations of the same surface area before and after a UV irradiation sequence show that the apparent heights of the molecules do not always change from high to low, but also occasionally from low to high (Figure 2 e, example marked by a blue circle). This is not surprising, as there is a finite probability that 365 nm irradiation induces *cis*–*trans* photoisomerization. According to a more quantitative analysis of the dynamic fluctuations between the two isomers, during a total change in *cis* coverage from *θ*_cis_(*t*_1_)=0.15 to *θ*_cis_(*t*_2_)=0.32 approximately 7 % of the *cis* isomers that were present on the surface at *t*_1_ changed to the *trans* state.

The isomers are not randomly distributed on the surface but seem to form clusters, especially after a longer period of irradiation (Figure 2 d). This was verified quantitatively by a statistical analysis of the spatial distributions in the STM images for *cis* coverages of 0.15 and 0.32 (Figure 3; for details, see the Supporting Information).

**Figure 3:**
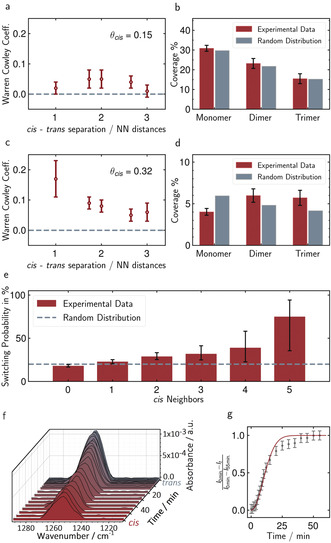
Quantitative analysis of photoswitching. a,c) Warren–Cowley coefficients of STM images at 0.15 and 0.32 *cis* coverage; b,d) the corresponding distributions of small ensembles. e) Switching probability as a function of neighboring molecules in the *cis* state (a *trans* molecule with 6 *cis* neighbors was not observed). The dashed line indicates the value expected for fully stochastic switching. f) IRRAS spectra of the C_phenyl_−O_methoxy_ stretching band of **1** on Au(111) during transition from the *trans*‐saturated state (gray) to the *cis*‐saturated state (red) upon 365 nm irradiation (at 10 μW cm^−2^). g) Corresponding temporal change of the normalized band intensity (symbols), which is a measure of the *cis* coverage, fitted by an Avrami growth model (solid line).

First, we determined the Warren–Cowley coefficients, which are a measure for the short‐range order.^22^ Second, we determined the coverages of small ensembles of *cis* isomers (monomers, dimers, trimers, surrounded by *trans* isomers) and compared those to the coverages obtained for a random distribution with the same *cis*/*trans* ratio on the surface.

At a surface coverage of 0.15 *cis* molecules, only slight deviations from a random distribution were found. The Warren–Cowley coefficients were all close to zero for the first five nearest neighbor distances (Figure 3 a), as expected for a statistical distribution. Furthermore, the coverages of small ensembles match those simulated for a random distribution within the statistical errors (Figure 3 b). In contrast, clear cluster formation was observed at 0.32 *cis* coverage. Here, the Warren–Cowley coefficients deviate significantly from zero (Figure 3 c) and the surface fraction of dimers and trimers is clearly enhanced at the expense of the fraction of monomers in comparison to a random distribution (Figure 3 d).

Both the Warren–Cowley coefficients as well as the increased coverage of multimers and lowered coverage of monomers indicates that neighboring adsorbates in the *cis* state accelerate the rate of *trans*–*cis* photoisomerization. This can also be demonstrated by direct analysis of the changes in isomeric state as a function of the state of the adjacent molecules (Figure 3 e). Here, we assume that only the direct neighbors in the adlayer affect the switching, and we determine from sequences of in situ STM images recorded in the same surface area, the probability for a molecule that is at time *t*_1_ in the *trans* state to be found at time *t*_2_ (that is, after irradiation) in the *cis* state. If the switching probability is independent of the state of the adjacent molecules, every molecule in the *trans* state will switch with the same probability, namely 20 % for a coverage change from *θ*_cis_(*t*_1_)=0.15 to *θ*_cis_(*t*_2_)=0.32 (Supporting Information).

However, the experimental data clearly shows an influence of neighboring *cis* isomers on the switching probability. Although the errors for molecules with 3 to 5 *cis* neighbors are high due to poor statistics (only a few switching events of these types are seen in the images), the trends toward higher *trans*–*cis* switching probability with an increasing number of neighboring molecules in the *cis* state are obvious. These studies, in which the temporal evolution of the isomerization state is directly monitored, also rule out other explanations of the cooperative effect, such as a decreased *cis*–*trans* back‐isomerization in the presence of *cis* neighbors.

The cooperative photoswitching observed on the atomic scale also manifests in the macroscopic photoisomerization kinetics of SAMs of **1**. We illustrate this by measurements with infrared reflection absorption spectroscopy (IRRAS), where we recorded the intensity of the stretching vibration of the terminal C_phenyl_−O_methoxy_ group attached to the azobenzene unit. Because of the rigid and well‐defined molecular structure, transitions between *trans* and *cis* states lead to defined orientation changes of this group relative to the surface, and thus, changes in the IR band's intensity, which allow monitoring of the photoisomerization.^19^, ^23^ Spectra of the C_phenyl_−O_methoxy_ stretch region during the UV‐induced transition from a *trans*‐saturated adlayer (gray), prepared by irradiation with 440 nm light, to the *cis*‐saturated state (red) are shown in Figure 3 f.

Upon irradiation with light of 365 nm the band intensity gradually decreased by nearly 60 %. These data allow to quantify the kinetics of the transition (for details, see the Supporting Information). Taking the different irradiation intensities into account, the time‐dependent changes in the *cis* coverage are in agreement with those in the STM images. Interestingly, the data do not show a simple exponential buildup of the *cis* state, as would be expected in the absence of interactions between the molecules, but a more sigmoidal dependence with a pronounced induction period (Figure 3 g). The observed *trans*–*cis* kinetics is well described by an Avrami‐type of 2D growth with instantaneous nucleation (solid line).^24^, ^25^ In this model, transition to the *cis* state occurs only at boundaries to existing *cis* molecules, starting from initial nuclei, which we identify with residual *cis* isomers in the *trans*‐saturated adlayer. Obviously, this behavior implies pronounced acceleration of the switching by neighboring *cis* isomers.

These collaborative effects cannot be explained by simple steric effects, because the large lateral distance of the molecules prevents direct contact between the functional groups (Figure 1 b). Furthermore, no indication for excitonic coupling was found in UV/Vis spectra of **1** SAMs (Figure S5). Thus, more subtle interactions have to be considered, which we examined in more detail by ab initio calculations (Supporting Information). Although addressing the photoexcited states in a molecular ensemble is computationally expensive and only feasible in simplified systems, qualitative insights can be obtained in this way. In a first approach, we considered a system of 14 vertical azobenzene units. The ground‐state interaction between a central *cis* and the surrounding *trans* isomers is mediated mainly by electrostatic and dispersion interactions between the methoxy groups of the bent *cis* and the N=N moiety of the *trans* azobenzenes. Consistent with the above statements, and due to the large separation between the azobenzenes, these interactions remain weak; only −5 kJ mol^−1^ for a *cis*–*trans* pair as compared to −0.2 kJ mol^−1^ for a *trans*–*trans* pair. Interestingly, the calculations reveal that the *trans*–*cis* interaction stabilizes the lone pair on the interacting *trans*. Consequently, the *n*–π* excitation on this *trans* isomer is the highest in the *n*–π* manifold (Figure 4). While the *n*–π* states are localized on individual photochromes, the π–π* states are delocalized on several photochromes.

**Figure 4:**
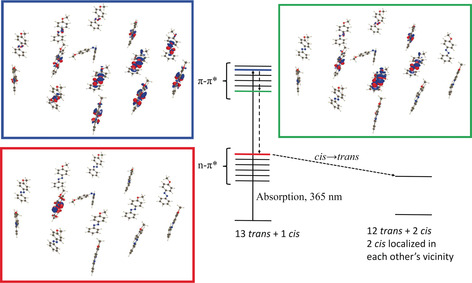
Excited states ordering and electron density differences for the model system of 13 *trans* and one *cis* isomers. The electron density differences (*ρ* ^ES^‐*ρ* ^GS^ determined on the ground‐state geometry) are shown for the π–π* with highest oscillator strength (blue highlight), lowest π–π* state (green highlight), and the first *n*–π* state (red highlight), which is localized on the interacting *cis*–*trans* pair.

Irradiation by UV light first induces the π–π* manifold, followed by a fast fall to the lowest π–π*, which is localized on azobenzenes surrounding the *cis*. In the subsequent de‐excitation to *n*–π*, the *n*–π* state localized on the *trans* isomer interacting with a *cis* isomer could act as a “trap”. Relaxation of this excited state eventually leads to *cis* clusters on the surface. This is, of course, a qualitative analysis and not all the photochromes would follow this pathway exactly. Nevertheless, such localization processes are well known in light harvesting systems and it was shown that they can also proceed in systems with large spatial separation.^26^, ^27^, ^28^

In a second, more local approach, we evaluated the impact of the surrounding photochromes—modeled as embedding point charges—on the *n*–π* excited state of a central *trans* isomer surrounded by six *trans* azobenzenes or by five *trans* and one *cis* isomer, respectively. At the CASPT2/ANO‐S level, the tilting of the C‐N=N‐C dihedral angle of the central photochrome is barrierless in the *n*–π* excited state, a statement that holds for both arrangements. However, the geometry optimization of the all *trans* case ends in a local minimum, while the same calculation for the *cis*‐containing cluster leads to a direct relaxation to the conical intersection. Of course, the molecule surrounded only by *trans* azobenzenes likely has enough kinetic energy in its excited state to escape from the shallow minimum and switch as well. Nevertheless, these different topologies of the potential energy surfaces hint that the surrounding molecules tune the excited state of the switch so as to tilt the probability toward the formation of *cis* clusters. Which of these two effects dominate is hard to determine, but both explain the observed *cis* clustering.

In conclusion, our results demonstrate that cooperative switching can occur even in freestanding photoswitches with spacings larger than 1 nm, where direct contact between the switchable groups can be excluded. Based on ab initio calculations, we attribute this observation to intermolecular electronic coupling, similar to light‐harvesting systems. Understanding and controlling such effects is of central importance for nanoscale applications of such switches; for example, for data storage or functional molecular machines. These may, on the one hand, require independent operation of the molecular units and thus suppression of cooperative switching. On the other hand, such collective phenomena may be exploited for the design of increasingly complex functional systems that involve molecular scale signal processing or cascade effects.

## Conflict of interest

The authors declare no conflict of interest.

## Supporting information

As a service to our authors and readers, this journal provides supporting information supplied by the authors. Such materials are peer reviewed and may be re‐organized for online delivery, but are not copy‐edited or typeset. Technical support issues arising from supporting information (other than missing files) should be addressed to the authors.

Supplementary
