# Supplementary material for: Observation of Collective Photoswitching in Free‐Standing TATA‐Based Azobenzenes on Au(111)
Source: Angew Chem Int Ed Engl. 2020 Jul 27;59(39):17192–6. doi: 10.1002/anie.202003797 (PMC7540444; doi:10.1002/anie.202003797)
Supplement: Supplementary file 1 — Supplementary [file ANIE-59-17192-s001.pdf]

Angewandte Chemie
Eine Zeitschrift der Gesellschaft Deutscher Chemiker

Supporting Information

Observation of Collective Photoswitching in Free-Standing TATA-Based Azobenzenes on Au(111)

*Talina R. Rusch, Alexander Schlimm, Nicolai R. Krekiehn, Tobias Tellkamp, Šimon Budzák, Denis Jacquemin, Felix Tuczek, Rainer Herges, and Olaf M. Magnussen**

anie_202003797_sm_miscellaneous_information.pdf

Supporting Information

Experimental Section

Sample Preparation

Compound **1**, which has a trioxabicyclo [2.2.2] unit implemented between the ethinyl spacer and the azobenzene group, was described in Ref. [1], where its synthesis and properties in solution are given in detail. The self-assembled monlayers were prepared on Au(111) single crystals with surface diameters of 10 mm and oriented within ±0.3° (MaTeck). For the IRRAS measurements, glass substrates with a 5 nm titanium adlayer and a 200 nm evaporated gold film from EMF corporation (Ithaca, NY) were used. All glassware used for the preparation was previously placed in 1/3 hydrogen peroxide mixed with 2/3 of sulfuric acid for at least 24 hours and carefully rinsed with Milli-Q water afterwards. The respective substrates were cleaned by flame annealing in butane gas and then immersed in 10 μM solutions of **1** in toluene (Merck, Uvasol) for 1 hour at 80 °C. Afterwards, the samples were rinsed with pure toluene, dried under ambient conditions and immediately thereafter, embedded into the STM or IRRAS setup.

IRRAS

The surface-adsorbed molecules were investigated using a Bruker VERTEX 70 FTIR spectrometer equipped with a polarization modulation accessory (PMA) 50 unit (Bruker Optik GmbH, Ettlingen, Germany). This instrument allows IRRAS and PM-IRRAS data to be recorded with a spectral range of 4000-800 cm^{-1}. IRRAS data were collected with a liquid-nitrogen-cooled MCT detector in a horizontal reflection unit for grazing incidence (Bruker A518). The sample chamber was purged with dry nitrogen before and during measurements. A deuterated hexadecanethiol SAM on Au(111) was used as a reference for the background spectrum for conventional IRRAS spectra. Each displayed spectrum is the average of 2048 measured spectra. A p-polarized beam at an incident angle of 80° to the surface normal was used for measurements. All spectra were recorded with 4 cm^{-1} resolution. PM-IRRAS data were collected with the PMA 50 accessory and a liquid-nitrogen-cooled MCT detector. The PEM maximum efficiency was set for the half-wave at 1750/3000 cm^{-1} for analysis of the area from 1400/2000 to 1000 cm^{-1}. All spectra were recorded with 4 cm^{-1} resolution. Processing of IRRAS and PM-IRRAS data was carried out using the OPUS software Version 6.5 (Bruker, Germany). Baseline correction of the resulting IRRAS data was performed by the rubber band method in an interactive mode. PM-IRRAS data were processed by the implicit removal of the Bessel function through manual baseline correction. For the *trans* - *cis* isomerization of compound adsorbed on Au(111) the prepared samples were irradiated within the spectrometer using LEDs (peak wavelength: 365 (± 9) nm or 440 (± 5) nm). For the reference measurements (I_{trans} and I_{cis}) of the *trans* and the *cis* saturated state Nichia NC4U133(T) LEDs (power dissipation: 12 W, luminous flux: 10 lm) were used. The IRRAS measurements were conducted at 294 K. The temperature of the sample was measured during the IRRAS measurements and irradiation experiments. No significant changes ($\Delta T = \pm 1$) were observed. At first, the sample was irradiated with light of 365 nm with 10 μW cm^{-2} for a certain period (1 min, 2 min, or 5 min) depending on the progress of the isomerization. Subsequently a PM-IRRA spectrum was measured for the determination of the C_{phenyl}-$O_{methoxy}$ stretch intensity. By repeating this procedure several times, the photoinduced *trans*/*cis*-isomerization was retraced by IRRA spectroscopy.

UV/Vis spectroscopy

The UV/Vis spectroscopy measurements were carried out with a Cary 4000 double-beam spectrometer (Varian Inc.) using the methodology of our previous studies.[2] The intensities of the spectrometer beam were $< 10^{-7}$ mW cm^{-2}, i.e., six orders of magnitude lower than those employed for the photoisomerization. The spectra for the **1** SAM were obtained on 10 nm thin (111)-oriented Au films on quartz under ambient conditions, using transmission geometry. The *trans-cis* and *cis-trans* photoisomerization was induced by irradiating the sample with 365 nm / 0.41 mW cm^{-2} (Nichia Corporation) and 455 nm / 0.38 mW cm^{-2} (Luxeon) LEDs, respectively. Photoisomerization of the molecules in solution was recorded with the same setup, using a cuvette with a thickness of 1 mm in place of the quartz sample holder. The spectra were measured at 1 nm resolution with an integration time of 0.1 s and a spectral bandwidth of 5 nm.

STM

The STM measurements were performed under ambient conditions with a PicoPlus STM (Agilent, Santa Clara, USA) and mechanically cut Pt/Ir (70:30) tips and carried out in constant current mode at tunneling currents of 30 to 50 pA and bias voltages of 200 to 400 mV.

STM images of highly oriented graphite were used for the in-plane calibration and the lateral drift was corrected with a dedicated software, developed by our group. The lattice constants and angles between rotational domains were analyzed using SPIP 6.7.6 (Image Metrology).

Two ultraviolet (365 nm) LEDs were used to irradiate the samples. The light was coupled via lenses into two 400 μm diameter quartz fibers. The ends of these fibers were mounted opposite of each other around the sample (Fig. S1), allowing to irradiate the sample. The intensity was calibrated using a photoactive sensor placed in the STM setup instead of the sample. The angle of incidence of the light from the quartz fibers can be adjusted and was set were between 7 and 10 degrees before each measurement. A these angles the intensity at the position of the tip was still high and shading by the tip small. Despite the shallow incident angles only a few switching

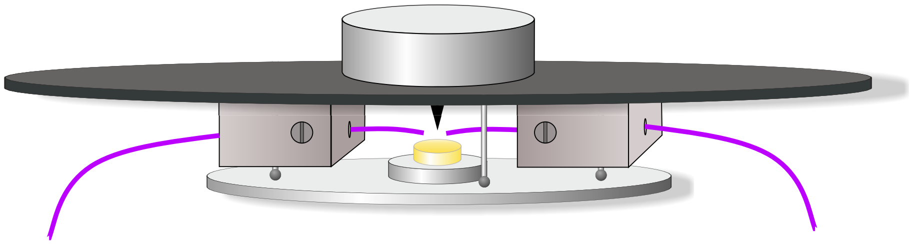

Figure S1: Schematic illustration of STM's UV irratiation setup, with the two quartz fibers shown in purple.

events could be observed when the tip was in tunneling contact during sample irradiation. This can be explained by (partial) shading of the imaged area by the tip. Because the typical tip radius is in the range of 1 μm (tunneling occurs via nanoscale protrusions on this sphere), the tip-sample distance in tunneling contact a few nm, and the typical size of the imaged area 50 nm, geometric shading occurs even at angles of 7-10 deg.

To ensure sufficiently exposure of the sample, the tip was withdrawn from the surface by approximately 800 nm between irradiation periods and approached again after exposure. In order to arrive at the same sample area again after approaching, the thermal drift must be low. Therefore, at the beginning of a sequence of measurements, the sample was first imaged with the measuring tip for one to two hours. This time was used to find a suitable area for the irradiation experiments. Since the adlayer is initially very homogeneous, it was important to find larger defects or long gold steps, which can be recognized even after changes in the isomeric states of the adsorbed molecules. Fig. S3 shows an unprocessed STM image from the evaluated measurement sequence. The step edge and the domain boundary served as distinct markers to recover the identical position on the sample surface after irradiation.

The STM images were drift corrected with SPIP (Scanning Probe Image Processor, Image Metrology A/S, Horsholm, Denmark) using a plug-in developed in our group. In addition, SPIP and Python 3.6 were used to reduce noise and increase contrast, especially in Fig. 2d and S4 b.

STM results

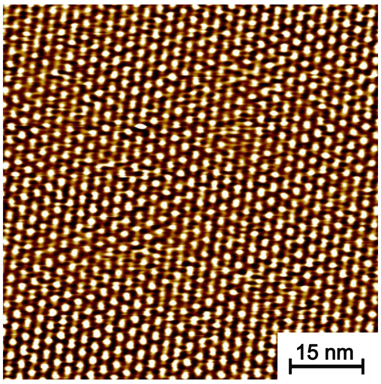

Figure S2: STM image of a self-assembled monolayer of **1** on Au(111) (35 x 35) nm^2 before irradiation.

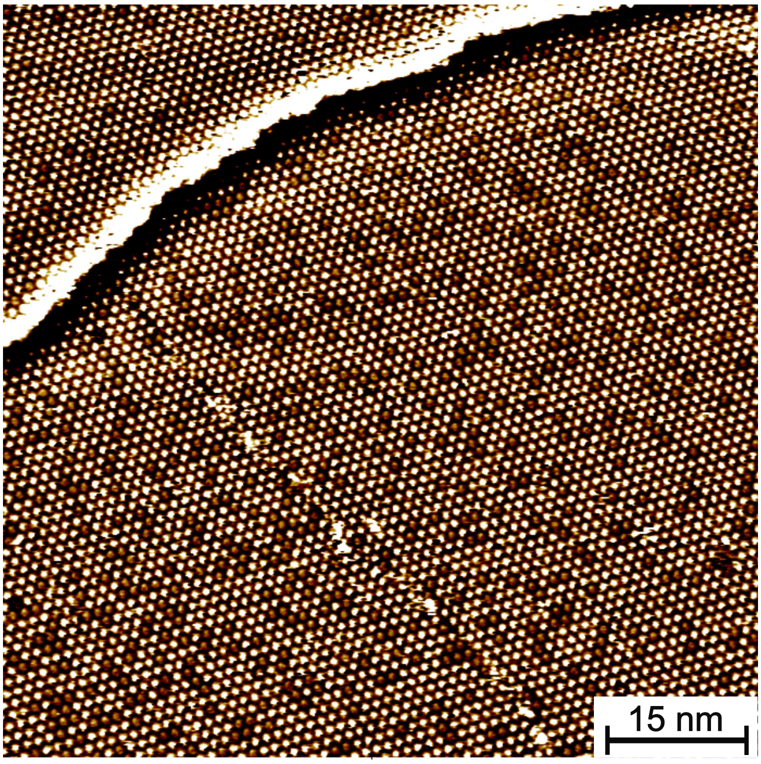

Figure S3: Unprocessed STM image of **1** on Au(111) after 28 minutes of irradiation with 365 nm at 6 $\mu W/cm^2$, (80 x 80) nm^2, I_t=30 pA, U_{bias}=0.3 V.

Quantitative Image Analysis

The statistical analysis focused on low to intermediate coverages, where most *cis* isomers are located in small clusters (monomers, dimers, trimers) embedded in a *trans* matrix. At higher coverages, a large variety of different *cis* clusters coexist, which differ in size and shape, leading to small numbers for each configuration and thus poor statistics. In addition, we molecular-resolution STM images were experimentally more difficult to obtain at high *cis* coverage, resulting again in low counting statistics.

The images were first transferred to a mathematical grid with a 1-0 matrix (Fig. S4a-d). Only sections that did not show rotational domains or gold steps were used for the quantitative analysis. Ensemble sizes, switching probabilities and Warren Cowley coefficients were calculated from these, using home-build software written in Python.

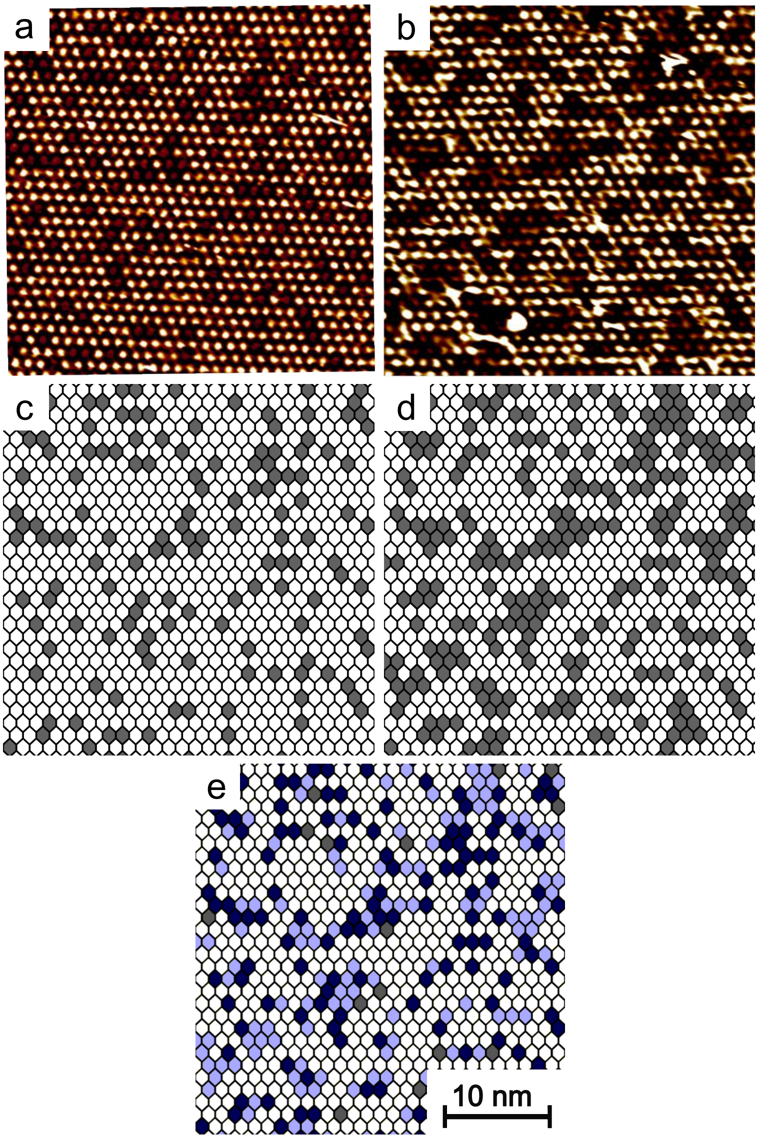

Figure S4: (a) STM image of a self-assembled monolayer of **1** on Au(111) after 28 minutes of irradiation with 365 nm at 6 μW/cm^2 and (b) after additional irradiation of 43 minutes with 365 nm at 6 μW/cm^2. (c, d) Data from (a) and (b) converted to a 1-0-matrix. The grey hexagons represent molecules in *cis* state and the white hexagons molecules in *trans* state. (e) Difference image of (c) and (d). The dark blue hexagons show *cis* and the white hexagons *trans* molecules from (c) that are unchanged. The light blue hexagons illustrate the molecules that isomerized from *trans* to *cis*, the grey hexagons illustrate the molecules that switched in the opposite direction.

Warren Cowley Coefficients
The Warren Cowley coefficients $\alpha(r)$ can be used as a measure of short-range order for binary mixed adlayers and were calculated as follows:[3]

$$\alpha(r) = 1 - \rho_{cis,trans}(r) \cdot \frac{1}{\theta_{cis}} \tag{1}$$

Here, $\rho_{cis,trans}(r)$ is the probability to find a molecule in *cis* state in a defined distance to a molecule in *trans* state, θ_{cis} is the coverage of *cis* isomers, and r is a particular lattice distance. In our study, $\alpha(r)$ was determined for r values of 1, $\sqrt{3}$, 2, $\sqrt{7}$, and 3 nearest neighbour distances). The Warren Cowley coefficients and ensemble sizes were determined using images from different surface areas. The errors were calculated by error propagation using the standard deviation from the mean.

Multimer Analysis
For the multimer analysis we generated random distributions for different ratios of $\frac{\theta_{cis}}{\theta_{trans}}$ and determined from those the coverage of small multimers. To keep the statistical error small, a large grid (1000×1000) was used. For verification purposes, we also calculated the average expected number of monomers, dimers, and trimers according to Ref. [4]. The results obtained by the simulation are in agreement with the calculated ones. The advantage of simulation is that the probability for large multimers can easily and quickly be determined.

Switching Probability
For the analysis of the switching probability as a function of the number of neighboring molecules in *cis* state, images taken in the same surface area before (t_1) and after (t_2) irradiation were used. As a result of this irradiation the *cis* coverage increased from $\theta_{cis}(t_1)$ to $\theta_{cis}(t_2)$. In the data analysis, first the molecules in *trans* state that at time t_1 have a certain number i (i = 1 to 6) of neighbors in *cis* state were identified. Of these n_i molecules k_i were found to have switched at t_2 to the *cis* state (graphically illustrated in Fig. S4e). From these data, the switching probability for molecules with i *cis* neighbors was determined as k_i/n_i.

Neighbours in *cis* state i	total number of molecules n_i	number of switching molecules k_i	ratio in %	90% confidence interval $[p_l, p_u]$ in %
0	2174	392	18.0	[16.7, 19.5]
1	826	188	22.8	[20.4, 25.5]
2	347	101	29.1	[25.3, 33.3]
3	86	28	32.6	[24.9, 41.3]
4	18	7	38.9	[22.7, 58.1]
5	4	3	75.0	[35.6, 94.2]
6	0	0		

Table S1: Switching probability as a function of the isomerisation state of the nearest neighbors, obtained from the statistical analysis of the STM data. To determine the switching probability, several image sections with a total of 4046 molecules were analysed.

The errors in Fig. 3e of the manuscript correspond to the 90% confidence interval and were calculated using the Wilson confidence interval for a binomial distribution [5]:

$$p_{u,l} = \frac{k_i + \frac{c^2}{2}}{n_i + c^2} \pm \frac{c\sqrt{n_i}}{n_i + c^2} \cdot \sqrt{\frac{k_i}{n_i} \cdot (1 - \frac{k_i}{n_i}) + \frac{c^2}{4n_i}} \tag{2}$$

with $p_{u,l}$ being the upper and lower boundary of the confidence interval and c the $1 - \frac{\alpha}{2}$ quantile of a standard normal distribution. A confidence level of $\alpha = 90\%$ was considered and therefore c = 1.644.

IRRAS Studies of the macroscopic kinetics

The macroscopic kinetics of the photoinduced *trans*-*cis*-isomerization of the **1** SAM was quantified in a similar way as in our previous study.[1] First, the integrated intensity I of the C_{phenyl}-$O_{methoxy}$ stretch band in the spectra was determined as a function of irradiation time t. Due to the surface selection rule, the IRRAS signal depends on the surface-normal component of the transition dipole vector of the methoxy group. Upon switching from the *trans* into the *cis* state, the orientation of the methoxy group relative to the surface changes, resulting in an intensity change. However, even in the *cis* state the transition dipole moment retains a (smaller) surface-normal component. Thus, even a SAM consisting only of *cis* isomers will exhibit a C_{phenyl}-$O_{methoxy}$ stretch band. To determine the *cis* fraction in the SAM we therefore subtract the band intensity of the photostationary state, which is reached at $t \leq 50s$, and normalize this value by the difference in intensity before and after UV irradiation, $I(t) - I(55min.)$.

This procedure is not fully exact, because even at 365 nm *cis*-*trans* reisomerization occurs at a low rate (as directly observable in the STM images), leading to a photostationary equilibrium with a *cis* isomer coverage $\theta_{cis} < 100\%$. For isolated molecules in solution, the *cis* fraction in the photostationary state is 85%, according to NMR measurements. For **1** SAMs an even higher *cis* fraction is expected, taking into account that θ_{cis} is high in the photostationary state and that the collective effects thus lead to an increase $k_{trans-cis}$ as compared to isolated molecules.

For the following modelling we ignore the deviation of θ_{cis} from 100%. This is justified, because (i) we are only interested in the qualitative behavior of $\theta_{cis}(t)$ and (ii) the collective effects mainly manifest at very low coverage. Here, the $\theta_{cis}(t)$ curve is not substantially affected by the saturation value. For the high coverage regime we do not expect a perfect agreement with the experimental data anyway, because of the simplicity of the employed models (see below).

To describe the IRRAS data shown in Fig. 3g, a number of different kinetic models were tested. Because a simple exponential function cannot reproduce the curve and the STM data indicate collaborative effects, several models that include adsorbate interactions were employed. These include (i) the model by Hauser et al.[6, 7], which describes the high-spin to low-spin relaxation in spin-crossover systems,[8] and essential results in a sigmoidal function and (ii-iv) models for the growth of two-dimensional films, based on the Avrami theorem, (ii) in the limit of instantaneous nucleation, (iii) in the limit of progressive nucleation, and (iv) in the general form with an intermediate island nucleation rate. Performing fits by these models, we found that the Avrami model with instantaneous nucleation described best the time-depend changes in *cis* coverage, determined from the IRRAS data. In this model, $\theta_{cis}(t)$ is given by:

$$\theta_{cis} = 1 - e^{-k \cdot t^2} \tag{3}$$

The best fit with this model is shown in Fig. 3g and corresponds to a value of the single parameter $k = 0.00547$. Although deviations from the measured data in the area of high *cis* coverage can be seen in the fitted curve, this is not unexpected in view of the simplifications in this model, e.g., the assumption of growth in form of circular islands growing centers, which is not the case according to the STM observations.

UV/Vis spectroscopy results

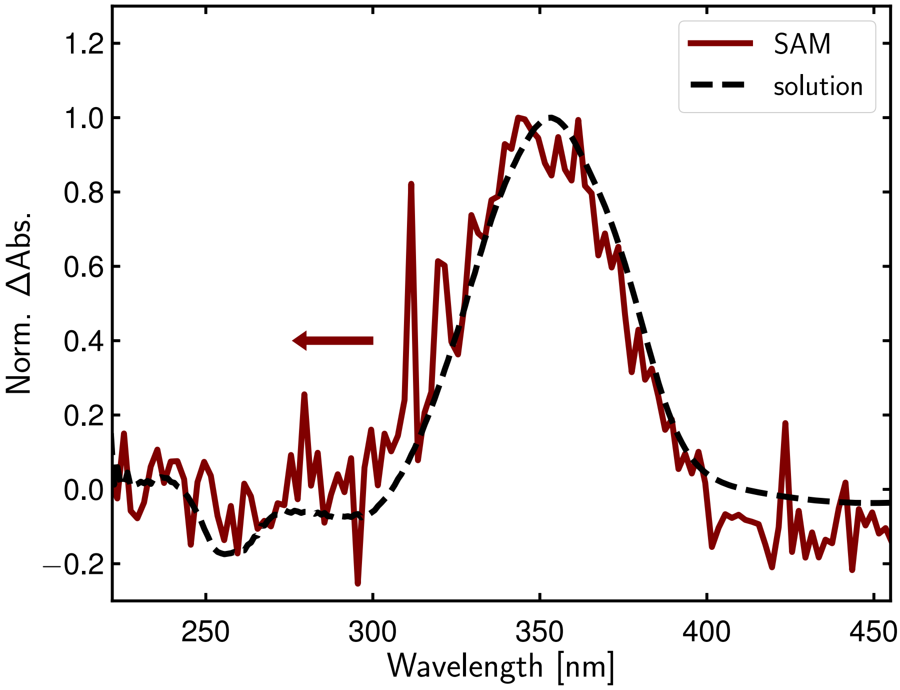

Figure S5: Normalized UV/Vis difference spectra (*trans* spectra minus *cis* spectra) for **1** in acetonitrile solution (dashed line) and as SAM on 10 nm thin Au substrate (solid line).The characteristic peak around 350 nm is caused by the $\pi - \pi^*$ transition of the azobenzene unit and centered at 353.5 nm for the free **1** molecule in solution and at 349.8 nm for the **1** SAM, respectively. Please note that the absorbances of the **1** SAM are only in the range 10^{-4}, leading to substantial noise in the spectra.

Theoretical Calculations

Methodology

To investigate collective effects in the photo-induced *trans-cis* isomerization of **1** adlayers, *ab initio* calculations of the photoexcited states for an ensemble of large organic molecules are required. These are computationally very expensive, as a large number of atoms and a multitude of excited states are involved and only possible for model systems. Thus, the calculations performed in this work provide qualitative insights into the mechanism rather than quantitative data that can be directly compared with the experiment.

Our methodology relied on Density Functional Theory (DFT) for the ground state geometry optimization. As the considered system is a large gold slab with p-methoxyazobenzene molecules grafted onto an anchoring platform, the AZB are well separated from the surface. In our model, we simulate this by a single hexagonal cell consisting of (at least) seven AZB. The distance between the dyes was set to reflect the experimental setup, i.e., 1.26 nm. The *trans* isomer was first optimized isolated in the gas phase and its geometry was used to create the cell. At such a distance the main interactions between the AZB molecules are of electrostatic and dispersion nature. We used the standard hybrid B3LYP functional [9] together with 6-31G(d) atomic basis set to perform the DFT calculations. While electrostatics is reasonably described with such a standard DFT approach, the dispersion effects were accounted for using an empirical correction, namely the so called DFT-D3-BJ correction [10]. To simulate the anchoring of the AZB the bottom hydrogen atom was frozen during the optimizations. Likewise, to prevent unrealistic tilting of the dyes, the nitrogen atom the closest from the surface was kept fixed. Test calculations revealed that freezing this nitrogen atom or the vicinal carbon atom led to essentially the same results. This set up allows the system to explore potential energy surface for rotations around H-N(C) axis but prevents it from "collapsing". All DFT calculations have been performed with Gaussian16 [11].

In order to gain additional insights into the origin of the interaction between the monomers we performed a decomposition of the interaction energy at the SAPT0/cc-pVDZ level of theory [12, 13] in PSI4 software [14].

To further investigate larger clusters of the *cis* isomers we performed calculation of the system with 14 AZB. This allows us to fully embed central 4 AZB and study their orientations. Since this system was too large to be treated by first principles, we used semi-empirical PM7 method [15] to obtain the structures and energies of these larger clusters. PM7 includes parameters for weak interactions.

For the excited state calculations, we opted for the Complete Active Space SCF (CASSCF) method [16]. The active space of 10 electrons in 8 orbitals was used and state-averaging over the three lowest states was always performed. These states are: the ground state, and both the $n-\pi^*$ and $\pi-\pi^*$ excited states. The active space includes the lowest π, π^* orbital pairs together with the two possible combinations of the nitrogen lone pairs. The dynamic electron correlation was accounted by multistate perturbation theory (CASPT2)[17]. To avoid intruder state problems the default value of IPEA (ionization energy, electron affinity) shift 0.25 au was introduced. Due to the size of the system, the multi-reference calculation was performed on the central AZB and all neighboring molecules were represented as a point charges. In these CASSCF and CASPT2 calculations the ANO-S-DZVP basis set was applied. All CAS calculations were performed with the OpenMolCas package [18].

Larger system of several *trans* monomers is needed for calculating exciton coupling effects. To this end we used geometry of the system with 14 AZB (see above) and ωB97X-D functional [19] in combination with 6-31G(d) basis set. From the 42 converged excited states, the lowest 14 correspond to the n-π^* states, the next 14 to the $\pi-\pi^*$ transitions. The n-π^* states are localized on individual AZBs, while $\pi-\pi^*$ states are linear combinations of excitations on different monomers.

Ground state characterization.

We started by calculating the relative energies of the different motifs in the ground electronic state. The DFT calculations clearly show a preference for two neighboring *cis* isomers compared to two separated *cis* isomers. As can be seen in the graph below (Figure S6), one would expect by simple addition that a central+peripheral *cis* block to be 41.5+49.8=91.3 kJ.mol^{-1} less stable than the all-*trans* cell in the ground electronic state. However, when two *cis* isomers are close, the structure is relatively stabilized by 83.5-91.3 = -7.8 kJ.mol^{-1}. In contrast, when the two *cis* isomers do not interact directly, the cooperative effect is much smaller, with 97.2-2x49.8=-2.4 kJ.mol^{-1}. Although these are relative total energies for the final photochromic states, this simulation shows a ther-

modynamic preference for the clustering of *cis* AZB, hinting that if they are formed, there is no thermodynamic reason that would make them unstable. In addition, in contrast to the all-*trans* cell in which the rotation of the monomers is unhindered at room temperature, two neighboring *cis* isomers cannot rotate freely. In fact, one can build two different relative orientations for the cell with two neighboring *cis* AZB: the first with parallel orientation of both *cis* and the second being the anti-parallel counterpart. Irrespective of these orientations, the cell with the neighboring *cis* isomers is stabilized by stronger dispersion interactions with the neighboring trans dyes (as compared to the all-*trans* case), with short contacts of less than 0.3 nm. This qualitatively explains the stabilization of -7.8 kJ.mol^{-1}.

In addition, we have considered the thermal inversion, which is the usually preferred ground

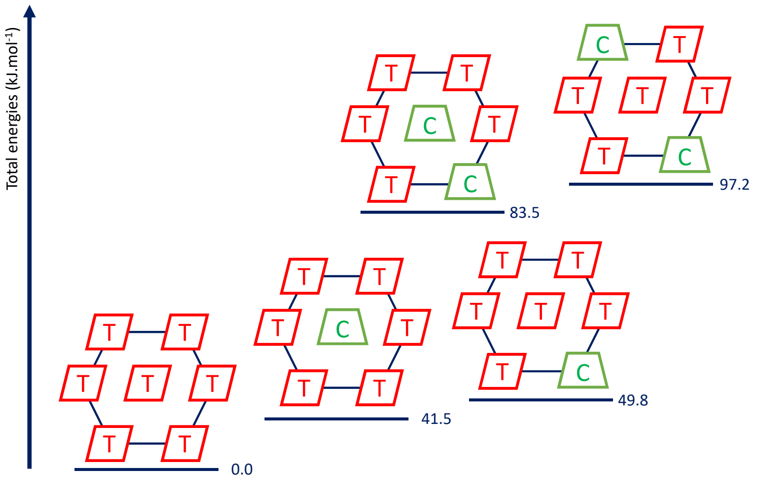

Figure S6: Relative energies of various clusters of *cis* and *trans* AZB.

state isomerization mechanism. The barrier for the *trans* to *cis* reaction is 179 kJ.mol^{-1} and does not depend on nature (*cis* or *trans*) of the surrounding molecules. The barrier for the thermal backreaction, relevant for our purposes, is of course smaller. In the case of the vicinal *cis* motif (2 in Figure S7), the barrier reaches 137 kJ.mol^{-1} which is larger than the one computed when only one central *cis* isomer back-transforms (131 kJ.mol^{-1}, case 1 on the same figure). These barriers indicate that the back reaction will likely be slow in all cases, especially with vicinal *cis* isomers.

Interaction energy decomposition.

The interaction energies between separate monomers in their ground electronic state are small, as a logical consequence of their large separation. We performed a decomposition of the interaction energy in order to gain more insights into its origins. The dimers for respective calculations were extracted from the cell with 5 *trans* and 2 vicinal *cis* monomers (Figure S8). Our results are summarized in the Table S2. The *cis*/*trans* interaction is stronger due to both electrostatics (e.g., point charges, dipole-dipole) and dispersion effects. The cell was dominated by *cis*/*trans* interactions so the *cis*/*cis* interactions generate only a small portion of interaction energy. The antiparallel *cis*/*cis* arrangement is dominated by dispersion.

The calculations above imply an interaction of the *cis* with a neighboring *trans* isomer, which likely depends on the conformations of the two molecules. Because of the rigid nature of **1**, the conformational degrees of freedom of *trans* and *cis* isomers in the SAM are largely limited to rotations of the molecules around their principal axis. The resulting changes in conformation are insignificant for the *trans* isomers, whereas rotation of the *cis* isomers can lead to larger variations in the position relative to neighboring molecules and hence different intermolecular interactions. To further investigate this aspect, we performed additional PM7 relaxed geometrical scans, in which the rotational orientation of the *cis* isomer was varied. We find that the energy is minimal when the *cis* isomer is oriented towards one of the neighboring *trans* isomers and that the bar-

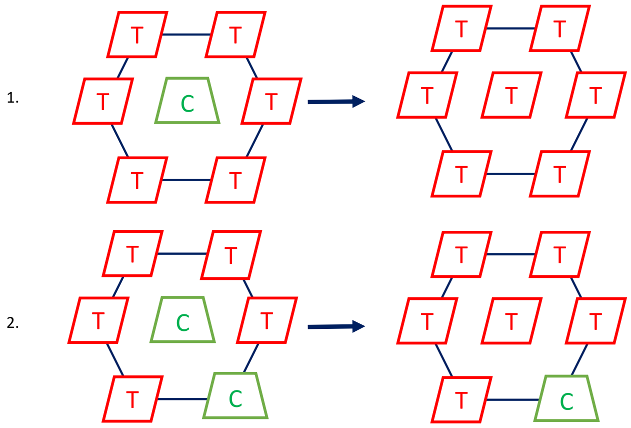

Figure S7: Ground state reactions studied at the DFT level.

Motif	Total interaction	Electrostatics	Exchange repulsion	Induction	Dispersion
Two *trans* face-face	0.3	0.4	0.0	0.0	-0.1
Two *trans* face-side	-0.2	0.0	0.0	0.0	-0.1
Two *cis* parallel	0.2	0.4	0.0	0.0	-0.2
Two *cis* antiparallel	-0.3	0.1	0.0	0.0	-0.4
cis-*trans*	-5.3	-3.2	7.9	-1.3	-8.7

Table S2: Ground state interaction energy between different motifs decomposed at SAPT0/cc-pVDZ level of theory. All values are in kJ.mol^{-1}.

rier for rotation to the next trans isomer is 5 kJ/mol, i.e., roughly twice of the thermal energy at room temperature. From a statistical point of view, the existence of an energetical minimum implies that the system will spend a larger portion of time in such arrangement, compared to other conformations (although rotation from one minimum to another is possible). Even though other arrangements are possible, the system will have a higher probability to be in this energetically most probable situation. We thus employ this geometry in our subsequent excited state calculations.

Excited state calculations.
Let us now turn to the excited states. The experimental setup required irradiation of the surface for several minutes with 365 nm photons. Several previous studies have shown that the photodynamics of azobenzene is complex, but the main reactive channel follows excited states cascade $S_2 \rightarrow S_1 \rightarrow S_0$, i.e., the initially populated $\pi - \pi^*$ excited state decays into the lowest n-π^* excited state of AZB that actually performs the photochemical reaction[20, 21]. Therefore, we considered photoreactions following this process here. We have performed calculations starting from the Franck-Condon region of the differently arranged cells (see Figures S8 and S9):

1. 1 central *trans* + 6 peripheral *trans* → 1 central *cis* + 6 peripheral *trans*
2. 1 central *trans* + 1 peripheral *cis* + 5 peripheral *trans* → 1 central *cis* + 1 peripheral *cis* + 5 peripheral *trans*

First, we have found that independently on the surrounding molecular environment the rotation around C-N=N-C dihedral angle is barrierless in the excited state and therefore can lead to the conical intersection directly. Nevertheless, it is worth to underline that this pathway is not systematically followed because other (larger) gradients are present at the FC point. These larger gradients may yield to a shallow excited state minimum corresponding to the inversion movement. For the photochemical reaction 1 of Figure S9 we observe that the switching *trans* AZB reached this local minimum while for 2 the system follows a direct path towards the conical intersection (see

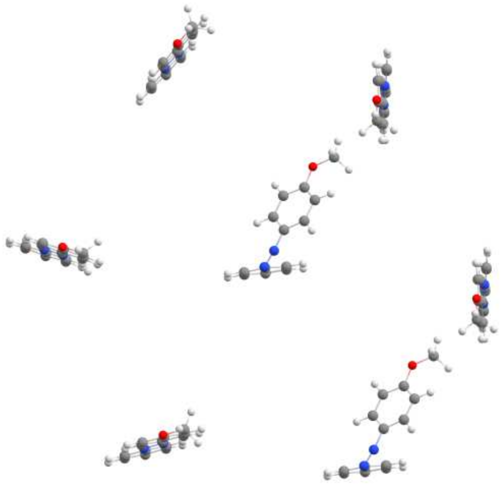

Figure S8: The cell consisting of two vicinal *cis* and 5 *trans* AZB isomers.

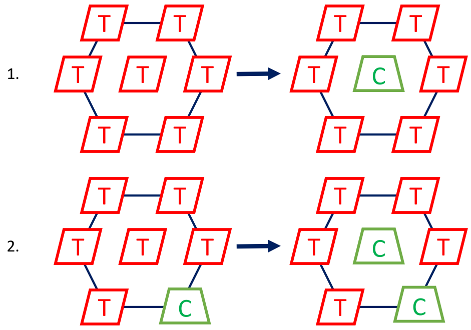

Figure S9: Schematic depiction of the studied photochemical reactions.

Figure S10). In order to judge if this is a direct effect of the embedding environment or an indirect effect from a reorientation of the central trans AZB in the ground state, we performed a geometry optimization of single AZB molecule starting from the same geometry as in case 2, but without the embedding charges. This optimization followed the path towards the local minimum. This supports the fact that the surrounding molecules, through their charge distribution, can influence the preferred relaxation path in the excited state, in the present case, leading to *cis* clusters. We observed the same behavior for the larger cell consisting of 13 trans and one *cis* isomer (see Figure S12).

On top of these CAS calculations performed on the minimal possible cell, we have also performed excited state calculations with TD-DFT theory on a larger cell containing 14 AZB (see Figure S11). The localization of n-π^* states was studied for this model, considering one *cis* isomer and 13 *trans* (see Figure S11b). In this latter arrangement, the highest - in energy - n-π^* state is localized on one of the *trans* isomer interacting with the already switched *cis* (though by a small margin). The excitation energies and oscillator strengths of first 28 states of this system are summarized in the Table S3 and the electron density difference for the highest excited state from the $n-\pi^*$ manifold is depicted in Figure S12. Therefore the initial energy accumulated in the $\pi-\pi^*$ state is preferably funneled to the (closest in energy) n-π^* state localized on the *trans* system vicinal to the *cis*, consistent with the formation of *cis* clusters.

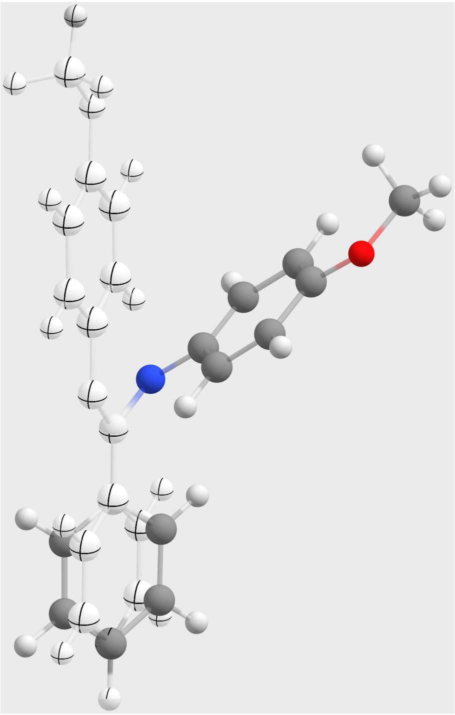

Figure S10: Overlay of the local minima structures reached on the excited state energy surface when the $n-\pi^*$ state is relaxed. White, crossed atoms depict structure optimized in all *trans* embedding. Standard color atoms show the structure optimized in the embedding with on *cis* isomer included. Note that the bent CNNC angle implies proximity of the ground state surface.

$n-\pi^*$	f	$\pi-\pi^*$	f
2.6092	0.0243	4.0135	0.0110
2.6356	0.0000	4.0159	0.0287
2.6358	0.0001	4.0187	0.0043
2.6362	0.0001	4.0217	0.0096
2.6367	0.0000	4.0245	0.0265
2.6393	0.0008	4.0312	0.0293
2.6407	0.0011	4.0358	0.1177
2.6408	0.0009	4.0377	0.0404
2.6411	0.0014	4.0474	0.0361
2.6415	0.0008	4.0542	0.0062
2.6420	0.0016	4.0706	0.2603
2.6423	0.0013	4.0822	0.5067
2.6443	0.0017	4.1102	10.3514
2.6668	0.0002	4.6434	0.3108

Table S3: Excitation energies (in eV) and oscillator strengths f of the lowest 28 states of the model system consisting of 13 *trans* isomers and one *cis*. In bold the highest $n-\pi^*$ and the most intense $pi-\pi^*$ state.

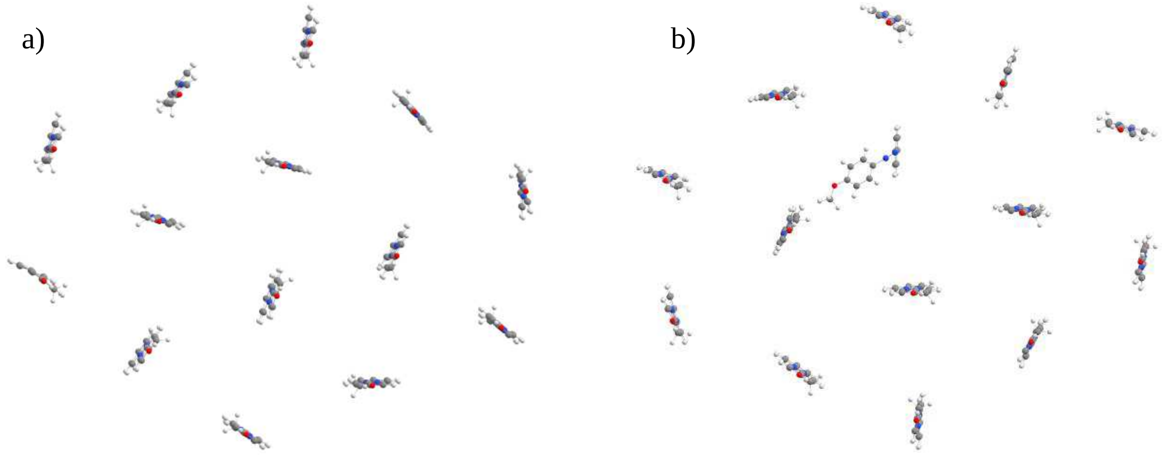

Figure S11: Large model system consisting of 14 photochromes: a) 14 *trans* isomers; b) 13 *trans* isomers and one *cis*. View from above.

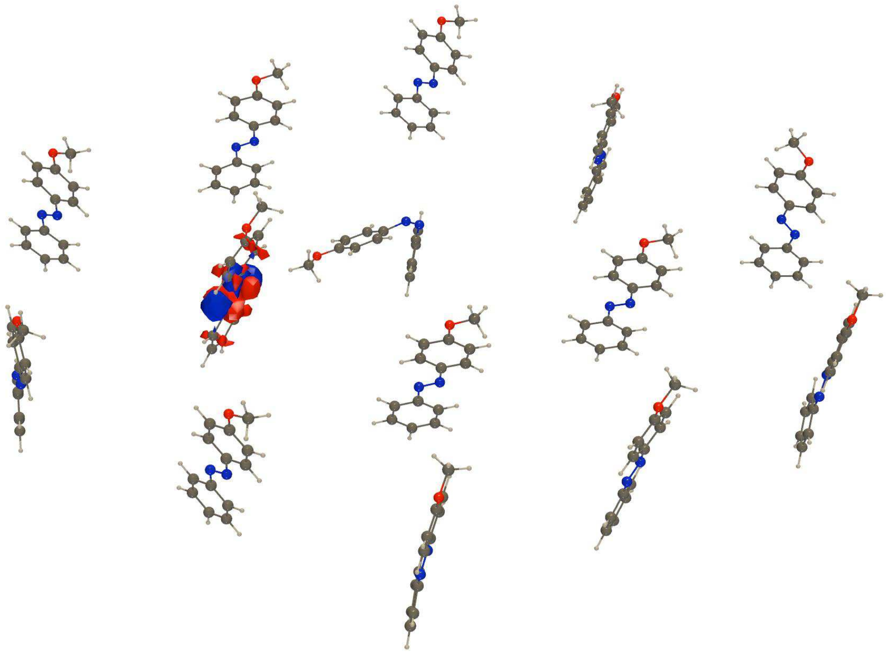

Figure S12: Electron density difference ($\rho^{ES} - \rho^{GS}$) determined on the Franck–Condon geometry for the highest n-π^* state. View from above.

Finally, we also checked whether a significant change of the absorption cross section can appear after isomerization of a central photoswitch. Up to now, we treated the whole slab (modelled as several azobenzenes) as one supersystem. In such an approach, the cross sections (TD-DFT oscillator strengths) of individual molecules are inaccessible as $\pi-\pi^*$ states are delocalized. The $n-\pi^*$ states are localized on individual molecules, but the experimental setup uses $\pi-\pi^*$ excitation (365 nm). To determine if the magnitude of the oscillator of the $\pi-\pi^*$ transition depends on the nature of the neighbouring photochromes, we used a different description of our system: we treated a central *trans* azobenzene molecule at TD-DFT level and modelled all the rest via molecular mechanics (we used MM charges to embed TD-DFT). With this strategy we can access the oscillator strengths of all transitions on the central molecule, but are neglecting the quantum mechanical interactions between the photochromes. Interestingly, we found that even at this simple level of theory, the energy levels follow the ordering we outlined above, i.e., the localization of the exciton on the *trans* azobenzene which is interacting with the *cis* azobenzene. This calculation also showed that the oscillator strengths of individual *trans* molecules that do not interact with a *cis* photochrome are slightly larger than in *trans* molecules directly interacting with a *cis* photochrome. However, the difference is less than 1.5% for all *trans* molecules and therefore not significant, considering the compromises made in the methodology to be able to compute the oscillator strengths of a single molecule. Thus, the cross sections differences between different *trans* molecules is not large enough to explain the observed effects.

References

[1] A. Schlimm, R. Löw, T. Rusch, F. Röhricht, T. Strunskus, T. Tellkamp, F. Sönnichsen, U. Manthe, O. Magnussen, F. Tuczek, R. Herges, *Angew. Chem. Int. Ed.* **2019**, *58*, 6574–6578.

[2] N. R. Krekiehn, M. Müller, U. Jung, S. Ulrich, R. Herges, O. M. Magnussen, *Langmuir* **2015**, *31*, 8362–8370.

[3] J. M. Cowley, *Journal of Applied Physics* **1950**, *21*, 24–30.

[4] M. F. Sykes, M. Glen, *J. Phys. A: MAth. Gen.* **1976**, *9*, 87–95.

[5] E. B. Wilson, *J. Am. Stat. Assoc.* **1927**, *22*, 209–212.

[6] A. Hauser, *Chemical Physics Letters* **1992**, *192*, 65–70.

[7] R. Hinek, P. Gutlich, A. Hauser, *Inorganic Chemistry* **1994**, *33*, 567–572.

[8] N. Moliner, L. Salmon, L. Capes, M. C. Muñoz, J.-F. Létard, A. Bousseksou, J.-P. Tuchagues, J. J. McGarvey, A. C. Dennis, M. Castro, R. Burriel, J. A. Real, *The Journal of Physical Chemistry B* **2002**, *106*, 4276–4283.

[9] A. D. Becke, *J. Chem. Phys* **1993**, *98*, 5648–5652.

[10] S. Grimme, S. Ehrlich, L. Goerigk, *Journal of Computational Chemistry* **2011**, *32*, 1456–1465.

[11] M. J. Frisch, G. W. Trucks, H. B. Schlegel, G. E. Scuseria, M. A. Robb, J. R. Cheeseman, G. Scalmani, V. Barone, G. A. Petersson, H. Nakatsuji, X. Li, M. Caricato, A. V. Marenich, J. Bloino, B. G. Janesko, R. Gomperts, B. Mennucci, H. P. Hratchian, J. V. Ortiz, A. F. Izmaylov, J. L. Sonnenberg, D. Williams-Young, F. Ding, F. Lipparini, F. Egidi, J. Goings, B. Peng, A. Petrone, T. Henderson, D. Ranasinghe, V. G. Zakrzewski, J. Gao, N. Rega, G. Zheng, W. Liang, M. Hada, M. Ehara, K. Toyota, R. Fukuda, J. Hasegawa, M. Ishida, T. Nakajima, Y. Honda, O. Kitao, H. Nakai, T. Vreven, K. Throssell, J. A. Montgomery, Jr., J. E. Peralta, F. Ogliaro, M. J. Bearpark, J. J. Heyd, E. N. Brothers, K. N. Kudin, V. N. Staroverov, T. A. Keith, R. Kobayashi, J. Normand, K. Raghavachari, A. P. Rendell, J. C. Burant, S. S. Iyengar, J. Tomasi, M. Cossi, J. M. Millam, M. Klene, C. Adamo, R. Cammi, J. W. Ochterski, R. L. Martin, K. Morokuma, O. Farkas, J. B. Foresman, D. J. Fox, *Gaussian~16 Revision C.01*, **2016**, Gaussian Inc. Wallingford CT.

[12] B. Jeziorski, R. Moszynski, K. Szalewicz, *Chemical Reviews* **1994**, *94*, 1887–1930.

[13] T. M. Parker, L. A. Burns, R. M. Parrish, A. G. Ryno, C. D. Sherrill, *The Journal of Chemical Physics* **2014**, *140*, 094106.

[14] R. M. Parrish, L. A. Burns, D. G. A. Smith, A. C. Simmonett, A. E. DePrince, E. G. Hohenstein, U. Bozkaya, A. Y. Sokolov, R. Di Remigio, R. M. Richard, J. F. Gonthier, A. M. James, H. R. McAlexander, A. Kumar, M. Saitow, X. Wang, B. P. Pritchard, P. Verma, H. F. Schaefer, K. Patkowski, R. A. King, E. F. Valeev, F. A. Evangelista, J. M. Turney, T. D. Crawford, C. D. Sherrill, *Journal of Chemical Theory and Computation* **2017**, *13*, 3185–3197.

[15] J. J. P. Stewart, *Journal of Molecular Modeling* **2013**, *19*, 1–32.

[16] B. O. Roos, P. R. Taylor, P. E. Sigbahn, *Chemical Physics* **1980**, *48*, 157 – 173.

[17] K. Andersson, P. Malmqvist, B. O. Roos, *The Journal of Chemical Physics* **1992**, *96*, 1218–1226.

[18] F. Aquilante, J. Autschbach, R. K. Carlson, L. F. Chibotaru, M. G. Delcey, L. De Vico, I. Fdez. Galván, N. Ferré, L. M. Frutos, L. Gagliardi, M. Garavelli, A. Giussani, C. E. Hoyer, G. Li Manni, H. Lischka, D. Ma, P. Malmqvist, T. Müller, A. Nenov, M. Olivucci, T. B. Pedersen, D. Peng, F. Plasser, B. Pritchard, M. Reiher, I. Rivalta, I. Schapiro, J. Segarra-Martí, M. Stenrup, D. G. Truhlar, L. Ungur, A. Valentini, S. Vancoillie, V. Veryazov, V. P. Vysotskiy, O. Weingart, F. Zapata, R. Lindh, *Journal of Computational Chemistry* **2016**, *37*, 506–541.

[19] J.-D. Chai, M. Head-Gordon, *Phys. Chem. Chem. Phys.* **2008**, *10*, 6615.

[20] M. Quick, A. L. Dobryakov, M. Gerecke, C. Richter, F. Berndt, I. N. Ioffe, A. A. Granovsky, R. Mahrwald, N. P. Ernsting, S. A. Kovalenko, *The Journal of Physical Chemistry B* **2014**, *118*, 8756–8771.

[21] C. Xu, L. Yu, F. L. Gu, C. Zhu, *Physical Chemistry Chemical Physics* **2018**, *20*, 23885–23897.
